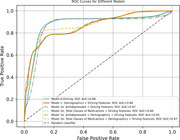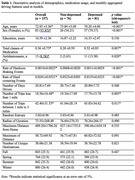# Identifying Major Depressive Disorder in Older Adults Through Naturalistic Driving Behaviors and Machine Learning

**DOI:** 10.1002/alz70857_102119

**Published:** 2025-12-24

**Authors:** Ganesh M. Babulal, Chen Chen, Yiqi Zhu, David C Brown, Matthew Blake, Jean‐Francois Trani

**Affiliations:** ^1^ Washington University School of Medicine, Saint Louis, MO, USA; ^2^ University of Johannesburg, Johannesburg, Gauteng Province, South Africa; ^3^ Knight Alzheimer Disease Research Center, St. Louis, MO, USA; ^4^ Washington University School of Medicine, SAINT LOUIS, MO, USA; ^5^ Washington University School of Medicine, St. Louis, MO, USA; ^6^ Washington University, St. Louis, MO, USA; ^7^ Natiobal conservatory of Arts and Crafts, Paris, NA, France

## Abstract

**Background:**

Depression in older adults is underdiagnosed and linked to adverse outcomes, including impaired driving and increased crash risk. As older adults increasingly rely on personal vehicles for independence, innovative, scalable screening methods are necessary. This study investigates whether naturalistic driving behaviors, captured via in‐vehicle GPS data, can identify major depressive disorder (MDD) in older adults using machine learning (ML) models.

**Method:**

A cohort of 157 older adults (mean age: 72.93 years; 52.87% female) participated, including 81 with clinically diagnosed MDD. Naturalistic driving data were collected over two years, analyzing metrics such as braking events, trip length, and driving entropy. Six models were developed using Extreme Gradient Boosting (XGBoost) to evaluate the predictive capacity of driving data alone and in combination with demographic and medication variables. Data preprocessing included hyperparameter tuning and 10‐fold cross‐validation to optimize model performance.

**Result:**

Driving features alone achieved an F1 score of 0.82, with an area under the curve (AUC) of 0.84, demonstrating high predictive accuracy. Models incorporating total medication classes with driving features showed the highest performance, with an F1 score of 0.81 and AUC of 0.86. Key predictive features included the rate of hard cornering, total medication use, and trip distances. Adding demographic variables (age, sex, and education) reduced model specificity, suggesting potential overfitting. Sensitivity analysis confirmed robustness when antidepressant users were excluded.

**Conclusion:**

Naturalistic driving data offers a promising avenue for detecting depression in older adults, with high specificity and recall. The integration of driving behaviors with medication data further enhances predictive accuracy, providing a functional and scalable approach for early identification. This study underscores the potential of AI‐driven tools to address diagnostic challenges in geriatric mental health, enabling timely interventions and improving public safety. Future research should explore integrating additional contextual data, such as cognitive assessments or real‐time physiological metrics, to refine model accuracy and expand clinical applications.